# Supplementary material for: Regulation of TRIF-mediated innate immune response by K27-linked polyubiquitination and deubiquitination
Source: Nat Commun. 2019 Sep 11;10:4115. doi: 10.1038/s41467-019-12145-1 (PMC6739404; doi:10.1038/s41467-019-12145-1)
Supplement: Supplementary file 1 — Supplementary Information [file 41467_2019_12145_MOESM1_ESM.pdf]

Supplementary Information

Regulation of TRIF-mediated innate immune response by K27-linked polyubiquitination and deubiquitination

Wu et al.

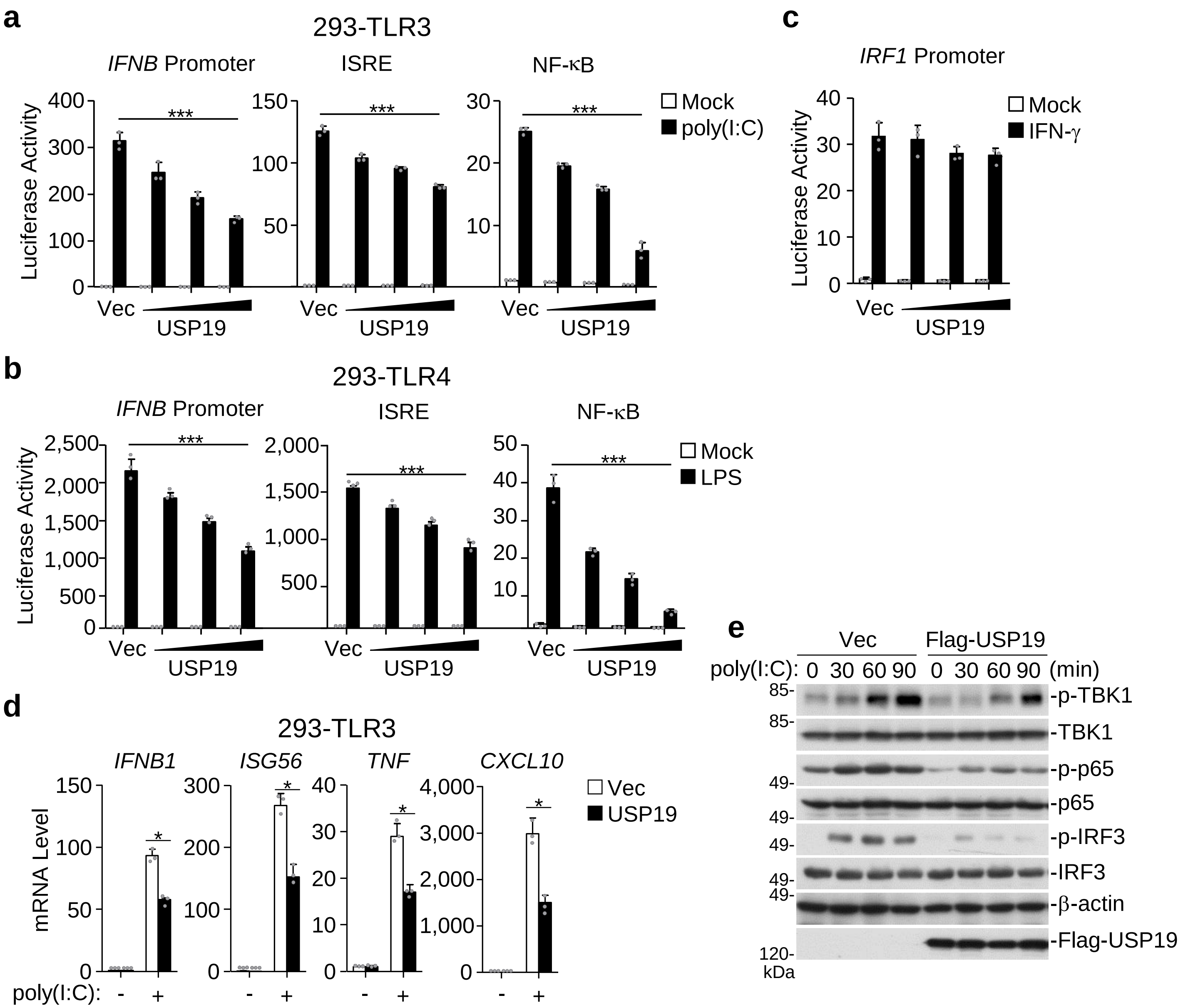

Supplementary Fig. 1. USP19 inhibits TLR3/4-triggered signaling.

(a, b) Effects of USP19 on poly(I:C)- and LPS-induced activation of IFN-β promoter, ISRE and NF-κB. The 293-TLR3 (**a**) or 293-TLR4 (**b**) cells were transfected with the IFN-β promoter, ISRE or NF-κB reporter plasmid as indicated and increasing amounts of USP19 plasmids. Twenty hours after transfection, cells were treated with poly(I:C) (50 μg/ml) (**a**), or LPS (100 ng/ml) (**b**), or left untreated for 8 h before luciferase assays were performed.

(c) Effects of USP19 on IFN-γ-induced activation of the IRF1 promoter. HEK293 cells were transfected with IRF1 promoter reporter and increasing amounts of USP19 plasmids. Twenty hours after transfection, cells were treated with IFN-γ (20 ng/mL) or left untreated for 8 h before luciferase assays were performed.

(d) Effects of USP19 on transcription of downstream genes induced by poly(I:C). The 293-TLR3 cells were transfected with USP19 plasmid for 20 h. The cells were then treated with poly(I:C) (100 μg/ml) or left untreated for 3 h before qPCR experiments were performed with the indicated gene primers.

(e) Effects of USP19 on poly(I:C)-induced phosphorylation of TBK1, p65 and IRF3. The 293-TLR3 cells were transfected with USP19 plasmid for 20 h. The cells were then treated with poly(I:C) (100 μg/ml) or left untreated for the indicated times before immunoblotting analysis with the indicated antibodies.

Graphs show mean ± SD; n = 3 independent samples in **a**, **b**, **c**, **d**, **f**. *P < 0.05, ***P < 0.001 (unpaired t test (**d**, **f**) or one-way ANOVA (**a**, **b**, **c**)). Data are representative of three experiments with similar results. Source data are provided as a Source Data file. Error bars represent standard deviation of the mean.

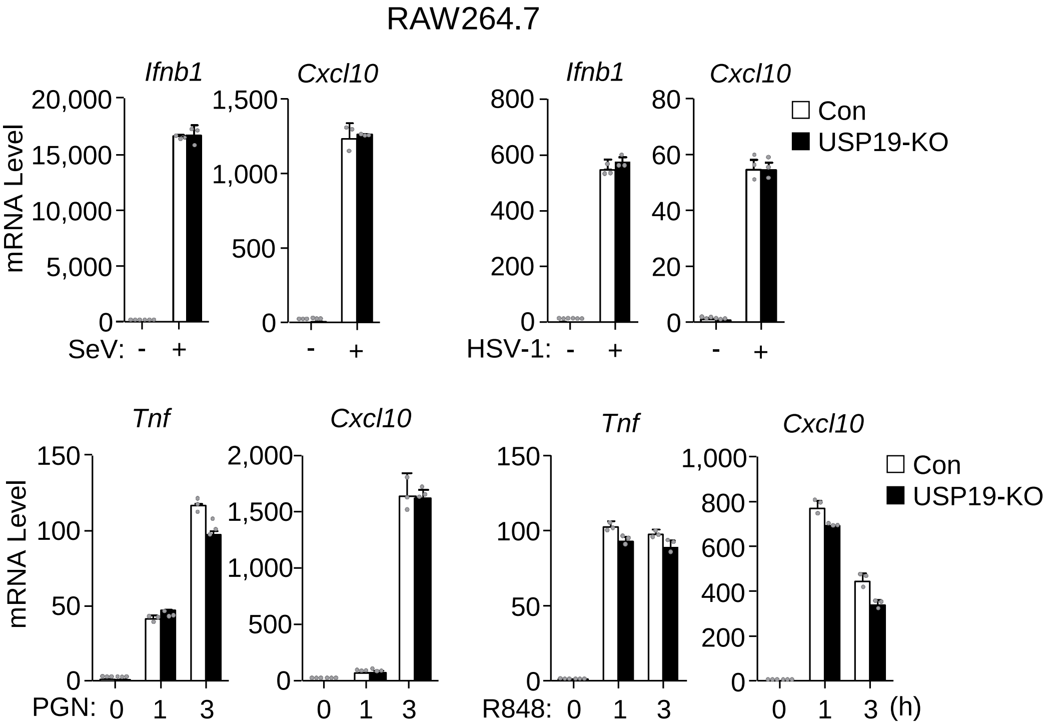

Supplementary Figure 2. Effects of Usp19-deficiency on transcription of downstream genes induced by various stimuli.

Usp19-KO RAW264.7 cells were generated by the CRISPR-Cas9 method. Usp19-KO and control cells were treated with SeV, HSV-1, PGN (20 μg/ml), R848 (20 nM) or left untreated for 3 h or the indicated times before qPCR analysis with the indicated gene primers. Graphs show mean ± SD; n = 3 independent samples. Data are representative of three experiments with similar results. Source data are provided as a Source Data file. Error bars represent standard deviation of the mean.

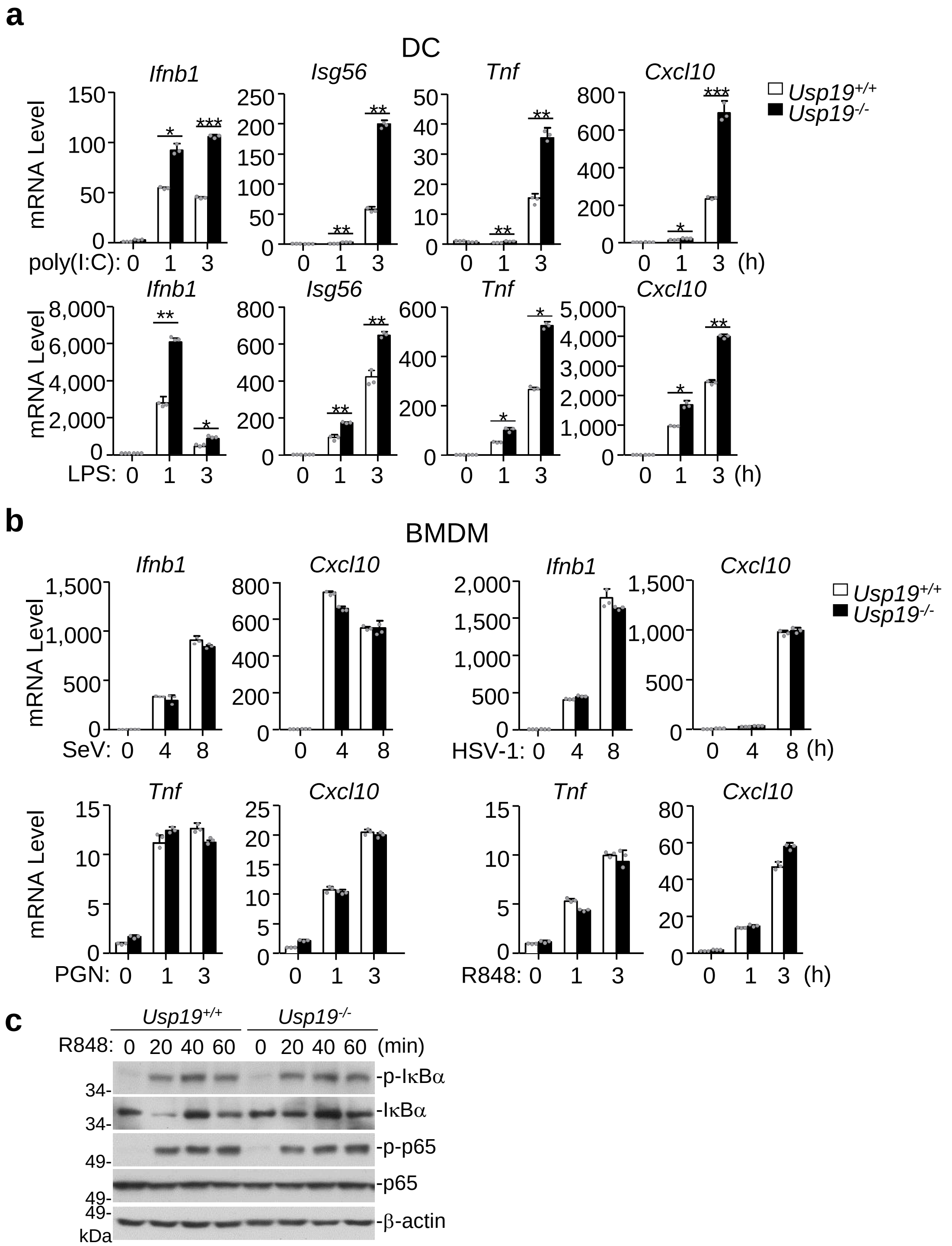

Supplementary Figure 3. Effects of USP19-deficiency on TLR3/4-mediated signaling.

(a) Effects of USP19-deficiency on poly(I:C)- and LPS-induced transcription of downstream genes. *Usp19*$^{+/+}$ and *Usp19*$^{-/-}$ DCs and MLFs were stimulated with poly(I:C) (50 μg/ml), LPS (50 ng/ml) for the indicated times before qPCR experiments were performed with the indicated gene primers.

(b) Effects of USP19-deficiency on SeV-, HSV-1-, PGN- and R848-induced transcription of downstream genes. *Usp19*$^{+/+}$ and *Usp19*$^{-/-}$ BMDMs were treated with SeV, HSV-1, R848 (20 nM) or PGN (20 μg/ml) or left untreated for the indicated times before qPCR experiments were performed with the indicated gene primers.

(c) Effects of USP19-deficiency on R848-induced phosphorylation of IκBα and p65. *Usp19*$^{+/+}$ and *Usp19*$^{-/-}$ BMDMs were treated with R848 (20 nM) or left untreated for the indicated times before immunoblotting analysis with the indicated antibodies.

Graphs show mean ± SD; n = 3 independent samples in **a**, **b**. *P < 0.05, **P < 0.01, ***P < 0.001 (unpaired t test). Data are representative of three experiments with similar results. Source data are provided as a Source Data file. Error bars represent standard deviation of the mean.

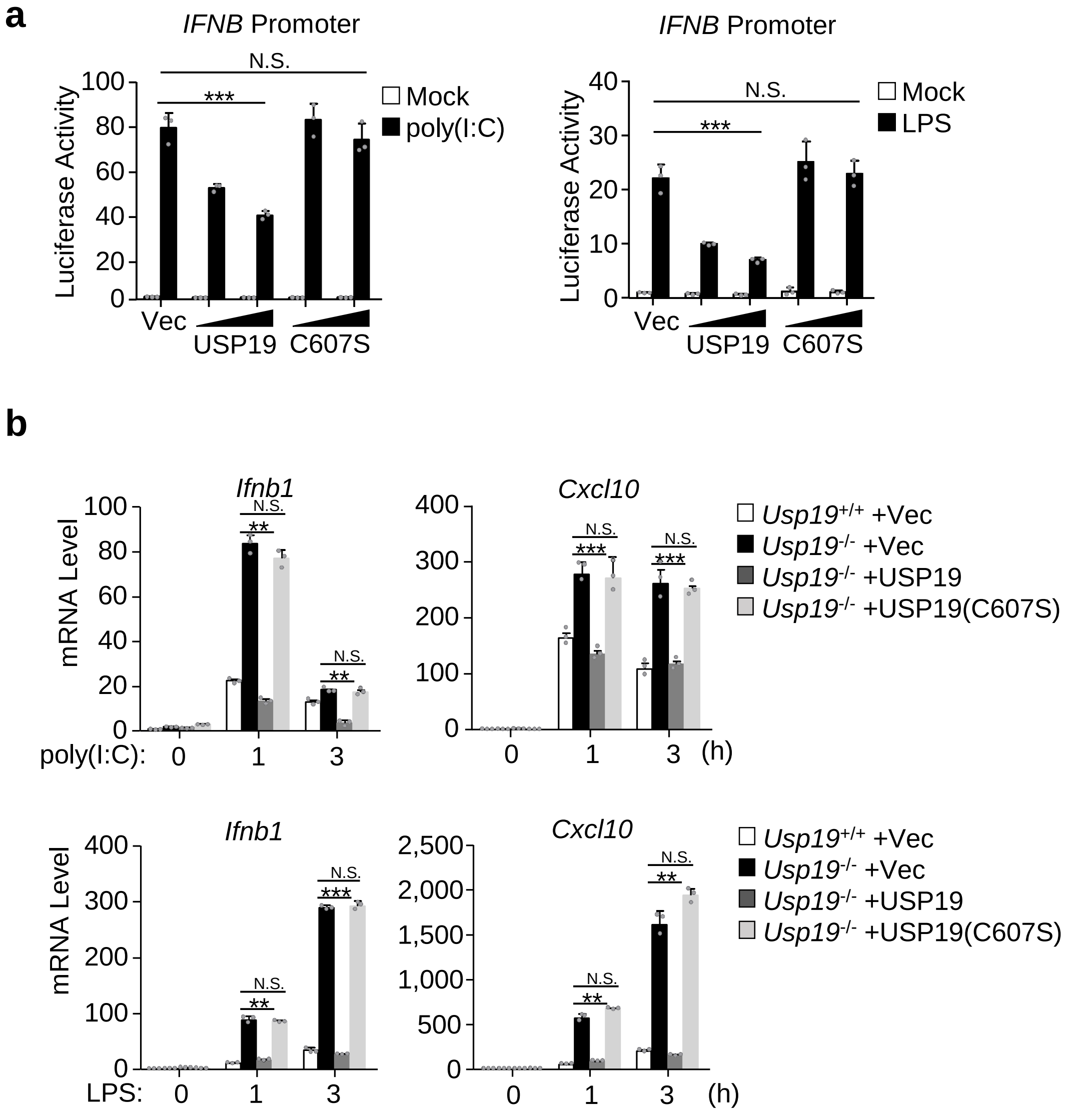

Supplementary Figure 4. Effects of USP19 and USP19(C607S) on TLR3/4-mediated signaling.

(a) Effects of USP19 or USP19 (C607S) on poly(I:C)- and LPS-induced activation of the IFN-β promoter. The 293-TLR3 or 293-TLR4 cells were transfected with IFN-β promoter reporter and increasing amounts of USP19 or USP19(C607S) plasmids. Twenty hours after transfection, cells were treated with poly(I:C) (50 μg/ml), LPS (100 ng/ml) or left untreated for 8 h before luciferase assays were performed.

(b) Reconstitution of USP19 inhibits poly(I:C)- and LPS-induced transcription of downstream genes. *Usp19*$^{-/-}$ MLFs were reconstituted with USP19 (WT) or USP19 (C607S). The reconstituted MLFs were stimulated with poly(I:C) (50 μg/ml) or LPS (50 ng/ml) for the indicated times before qPCR experiments.

Graphs show mean ± SD; n = 3 independent samples. **P < 0.01, ***P < 0.001 (unpaired t test (**b**) or one-way ANOVA (**a**)). N.S., not significant. Data are representative of three experiments with similar results. Source data are provided as a Source Data file. Error bars represent standard deviation of the mean.

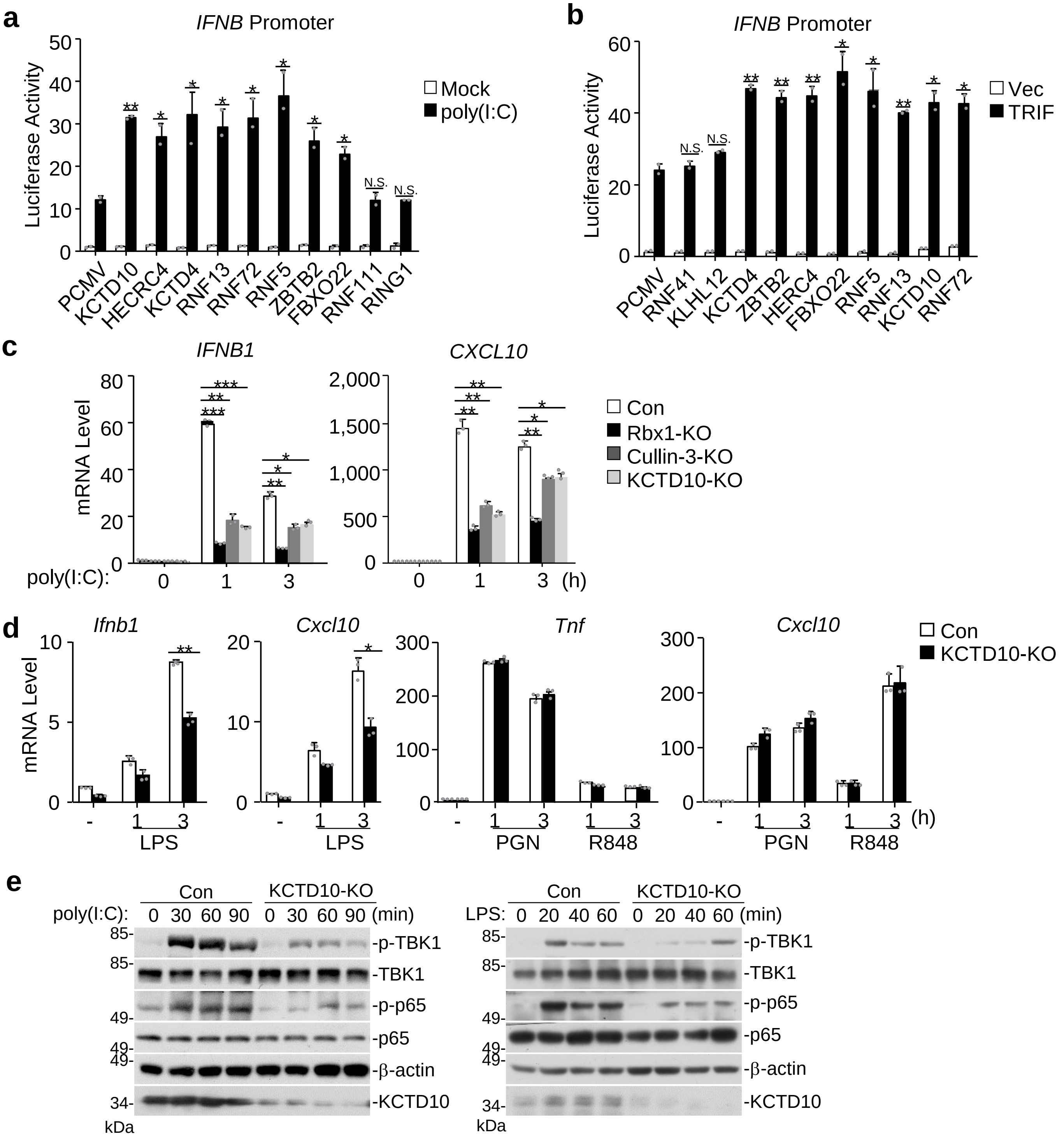

Supplementary Figure 5. Effects of various ubiquitin-related proteins on TLR3- and TRIF-mediated signaling.
(a) The 293-TLR3 cells (5x10^4) were transfected with the IFN-β promoter reporter (20 ng) and expression plasmids (50 ng each) encoding the indicated ubiquitin-related enzymes for 20 h. Cells were then treated with poly(I:C) (50 μg/ml) or left untreated for 10 h before luciferase assays were performed. **(b)** The 293-TLR3 cells (5x10^4) were transfected with the IFN-β promoter reporter (20 ng) and expression plasmids (50 ng each) for TRIF and the indicated ubiquitin-related enzymes for 20 h before luciferase assays were performed.
(c) Effects of deficiency of Cullin-3-Rbx1-KCTD10 complex on transcription of downstream genes induced by poly(I:C). Rbx1-, Cullin-3- or KCTD10-deficient and control 293-TLR3 cells were treated with poly(I:C) (100 μg/ml) or left untreated for the indicated times before qPCR analysis. **(d)** Effects of KCTD10-deficiency on LPS-, PGN- and R848-induced transcription of downstream genes. The control or KCTD10-deficient RAW264.7 cells were treated with LPS (100 ng/ml), PGN (20 μg/ml), R848 (20 nM) or left untreated for the indicated times before qPCR analysis. **(e)** Effects of KCTD10-deficiency on poly(I:C)- and LPS-induced phosphorylation of TBK1 and p65. The control or KCTD10-deficient 293-TLR3 or RAW264.7 cells were treated with poly(I:C) (100 μg/ml) or LPS (100 ng/ml) for the indicated times before immunoblotting analysis with the indicated antibodies. Graphs show mean ± SD; n = 2 independent samples in **a**, **b**; n = 3 independent samples in **c**, **d**. *P < 0.05, **P < 0.01, ***P < 0.001 (unpaired t test); N.S., not significant. Data are representative of at least two experiments with similar results. Source data are provided as a Source Data file. Error bars represent standard deviation of the mean.

Figure 1a

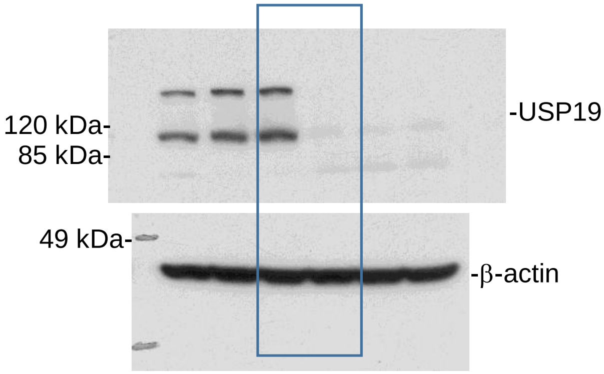

Figure 1b

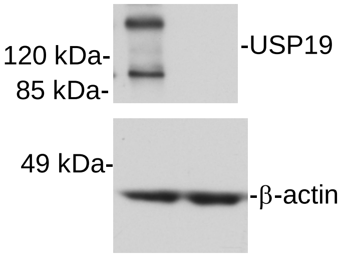

Figure 1c

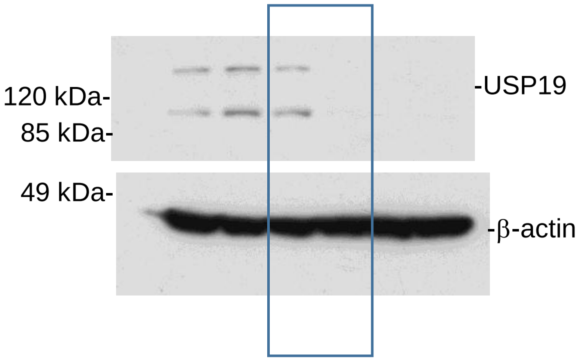

Supplementary Figure 6. Full size immunoblots of cropped blots in the main manuscript figures. Full size immunoblots of cropped blots for Fig. 1a, 1b and 1c.

Figure 2b

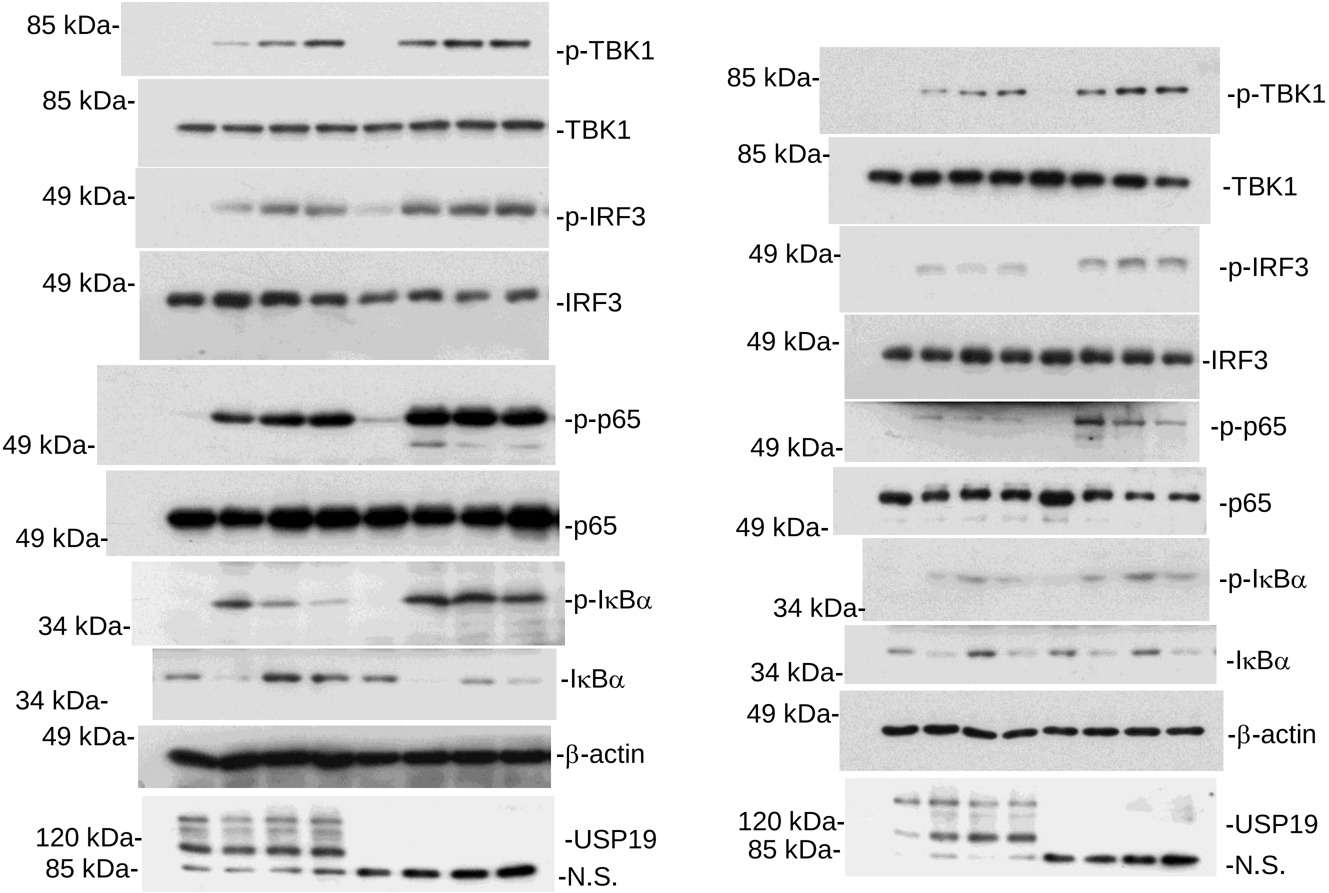

Supplementary Figure 7. Full size immunoblots of cropped blots in the main manuscript figures. Full size immunoblots of cropped blots for Fig. 2b.

Figure 4b

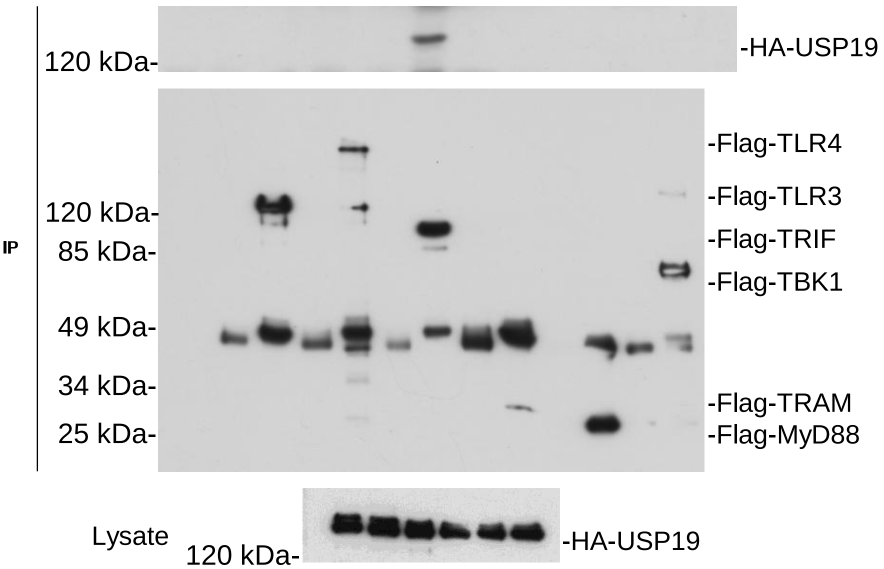

Figure 4c

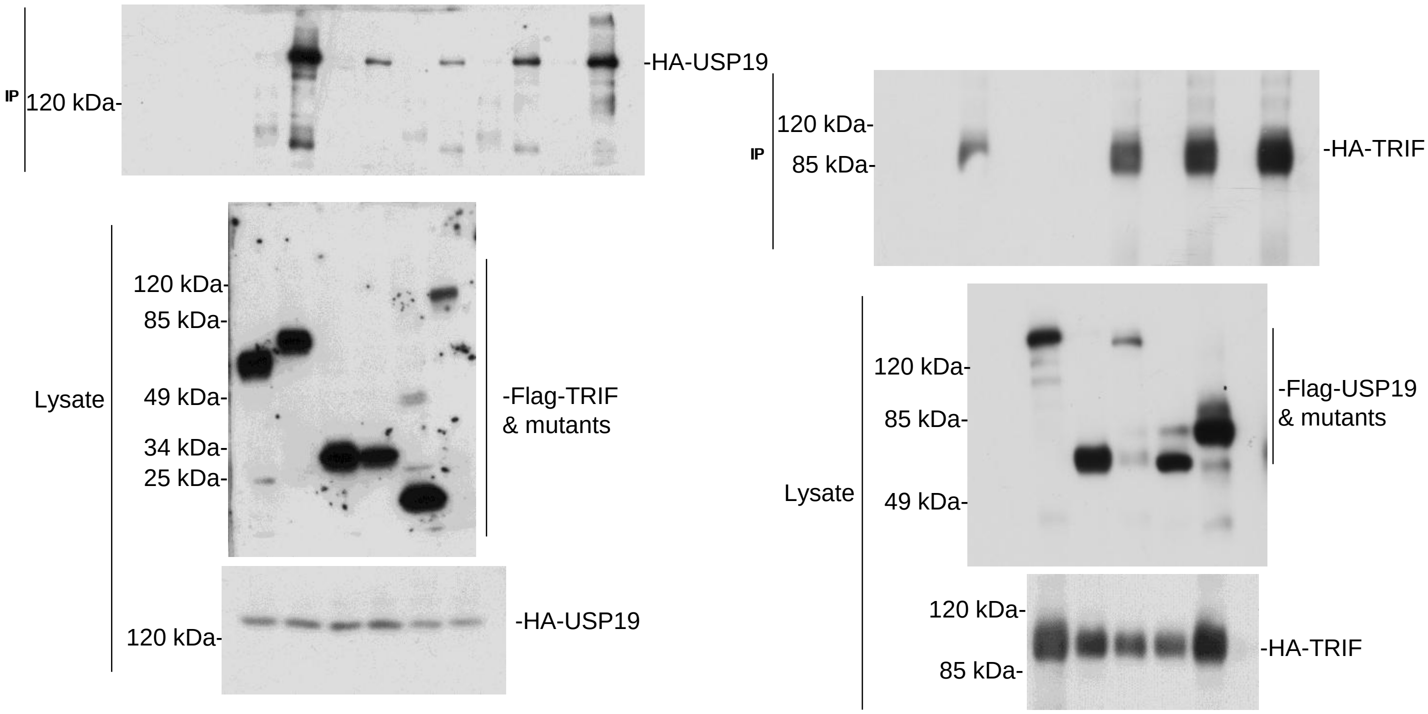

Figure 4e

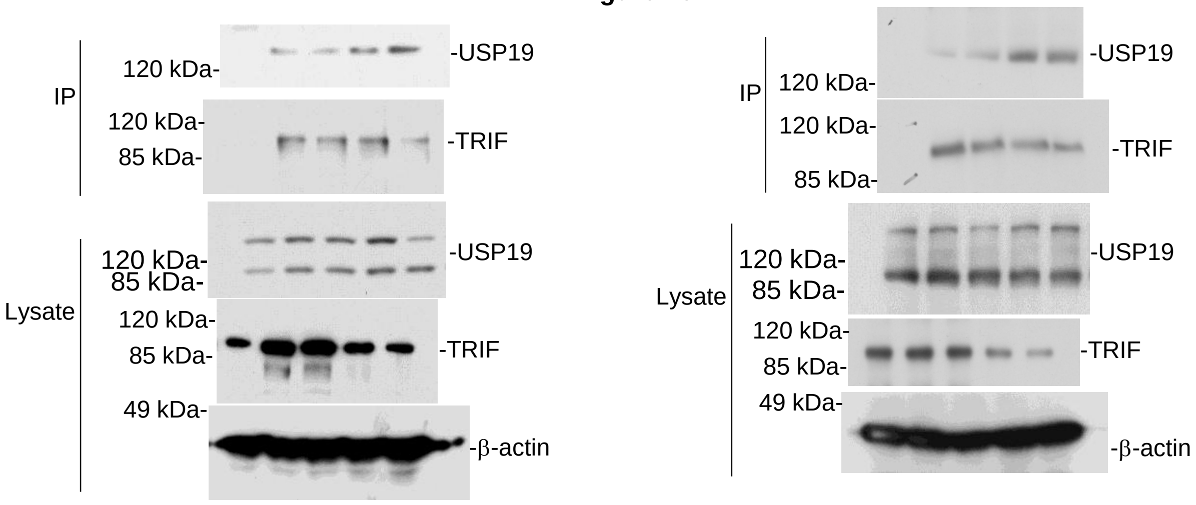

Supplementary Figure 8. Full size immunoblots of cropped blots in the main manuscript figures. Full size immunoblots of cropped blots for Fig. 4b，4c and 4e.

Figure 5a

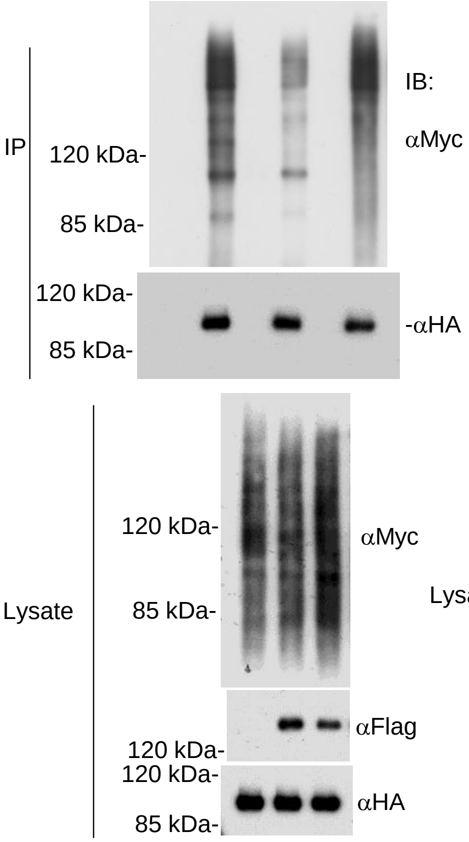

Figure 5b

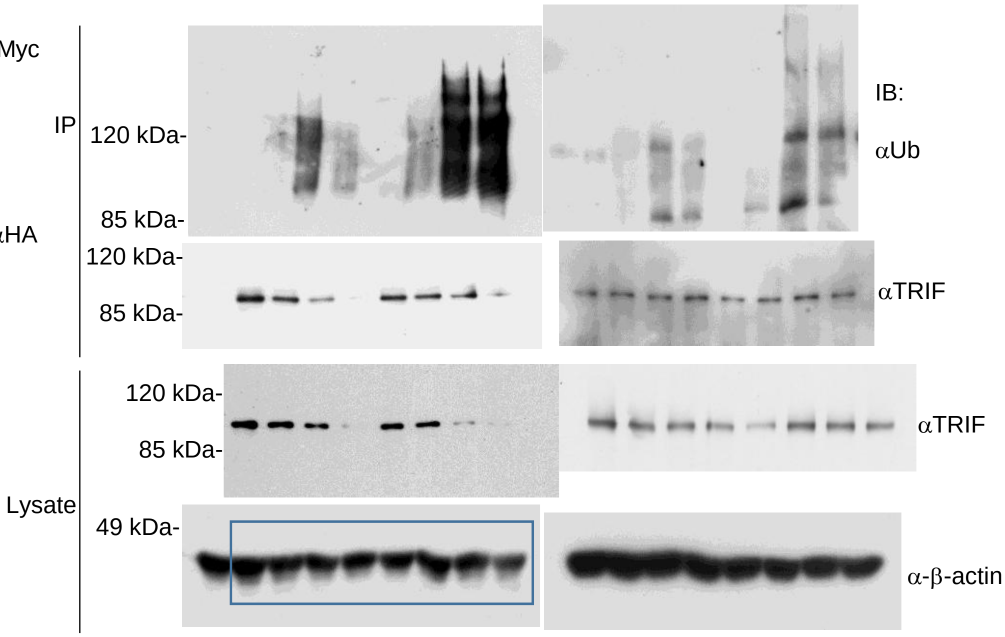

Figure 5c

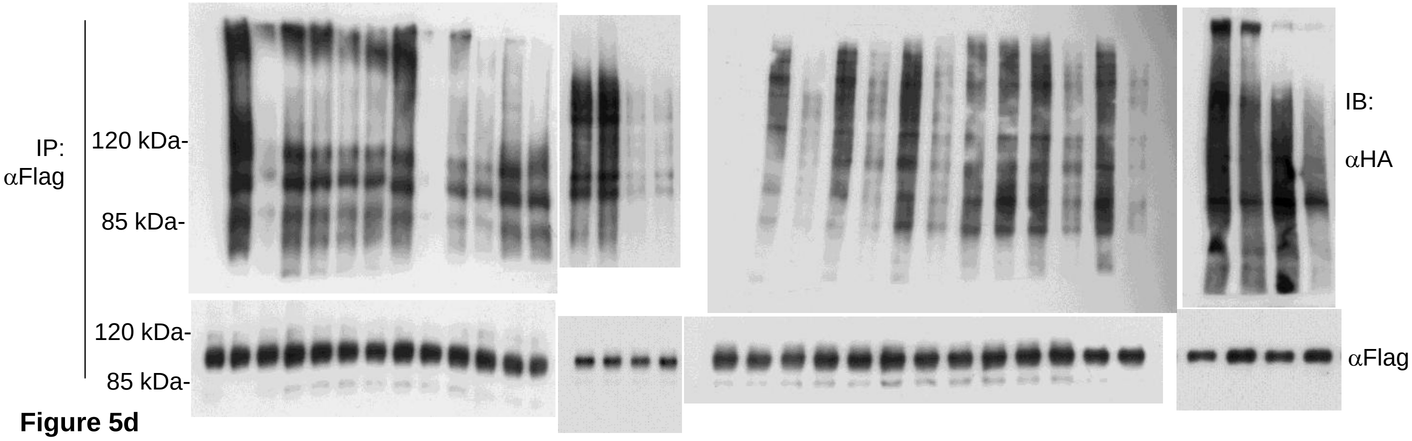

Figure 5d

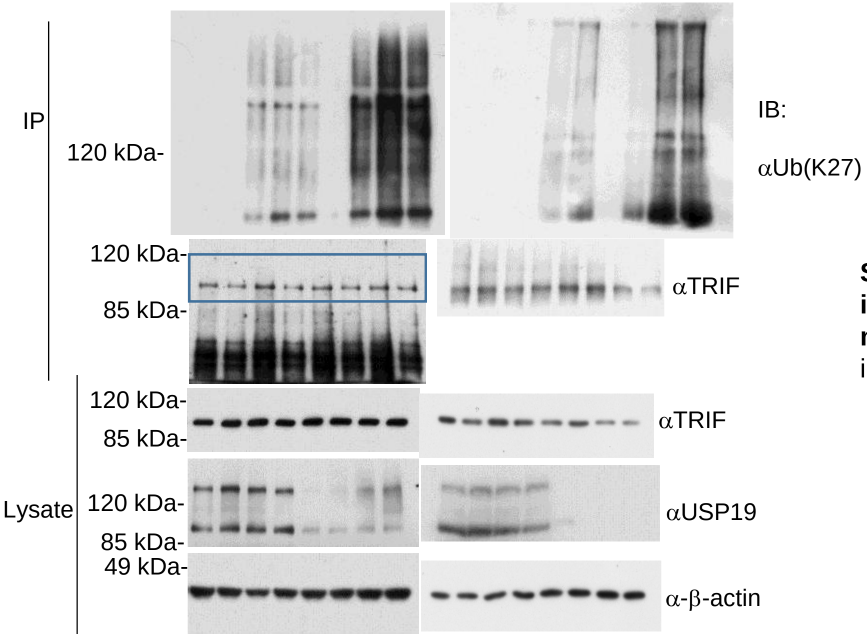

Supplementary Figure 9. Full size immunoblots of cropped blots in the main manuscript figures. Full size immunoblots of cropped blots for Fig. 5a-d.

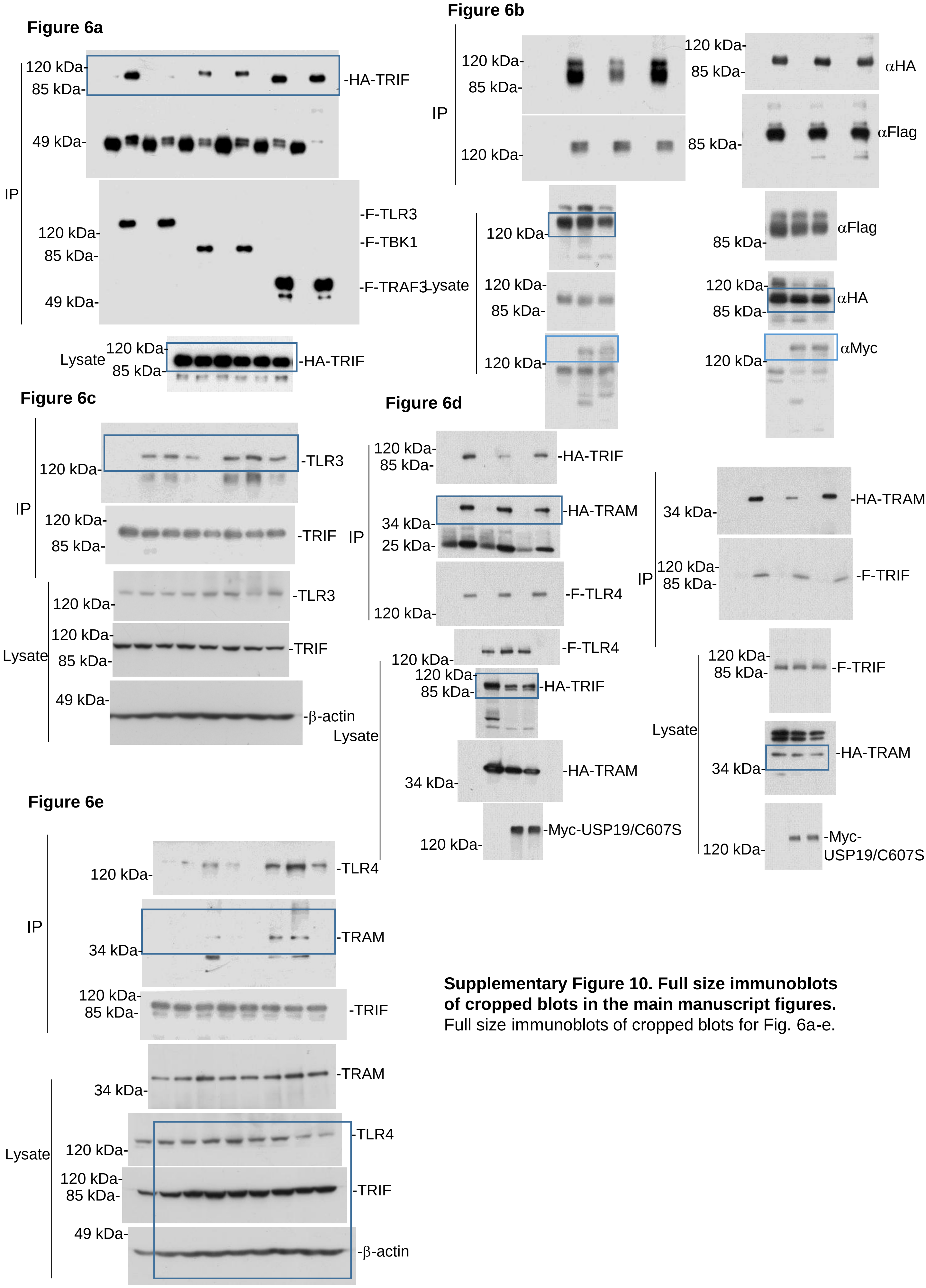

Supplementary Figure 10. Full size immunoblots of cropped blots in the main manuscript figures. Full size immunoblots of cropped blots for Fig. 6a-e.

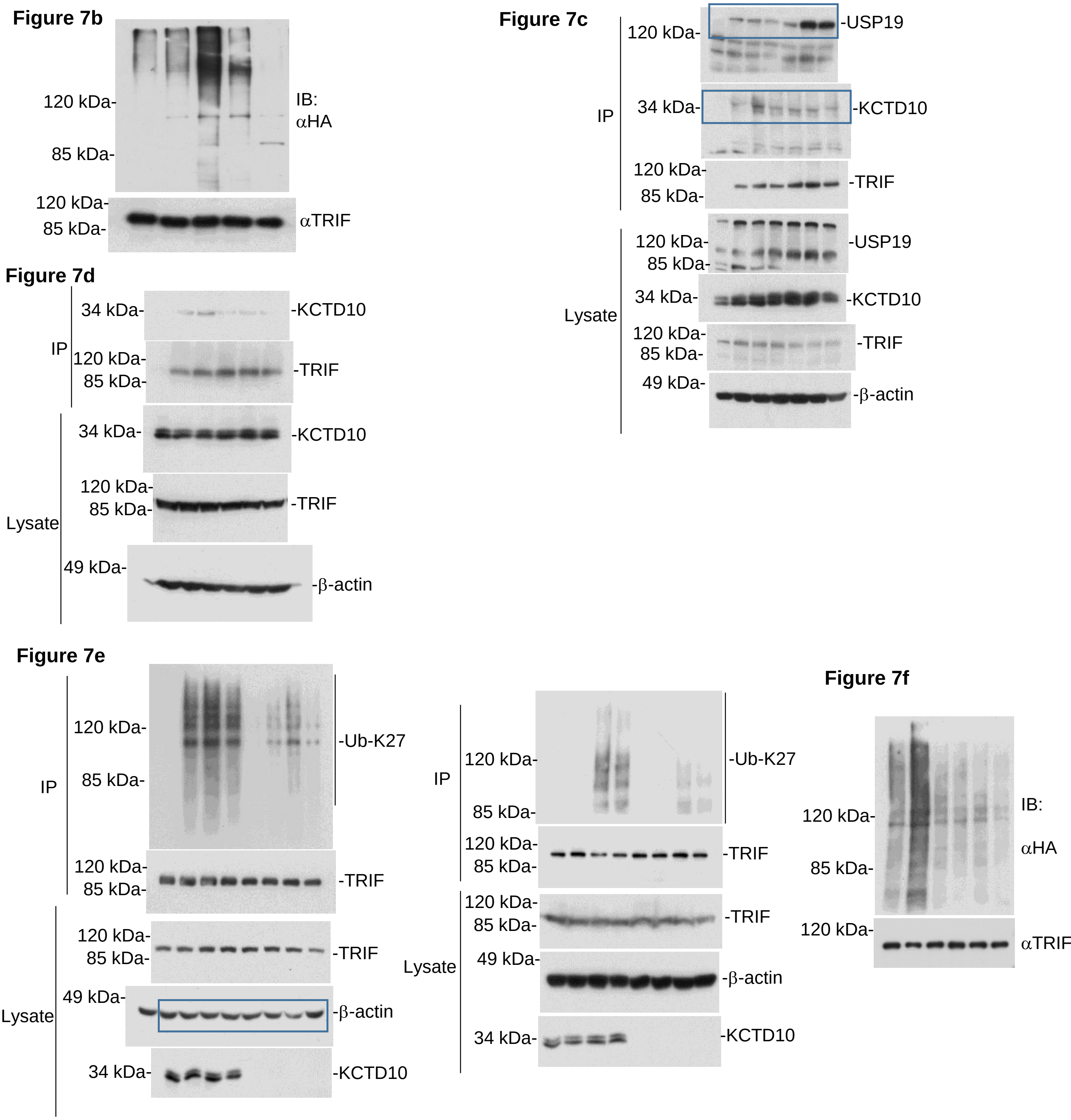

Supplementary Figure 11. Full size immunoblots of cropped blots in the main manuscript figures.
Full size immunoblots of cropped blots for Fig. 7b-f.

Figure 8c

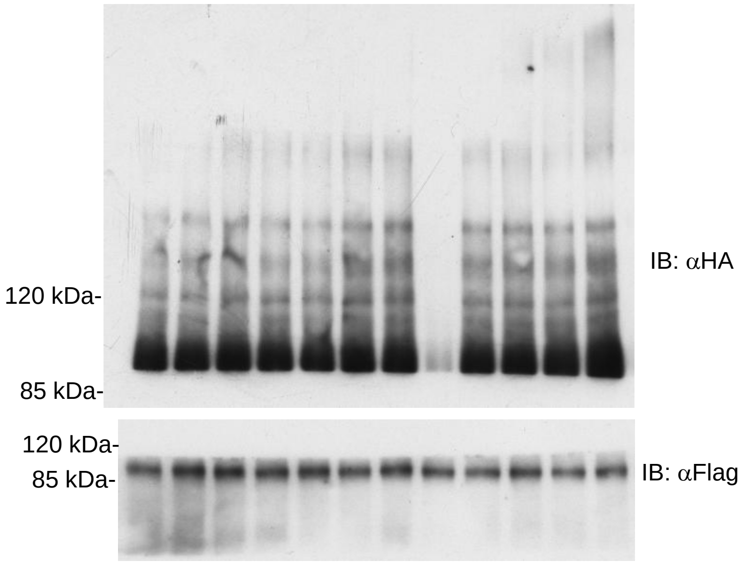

Figure 8d

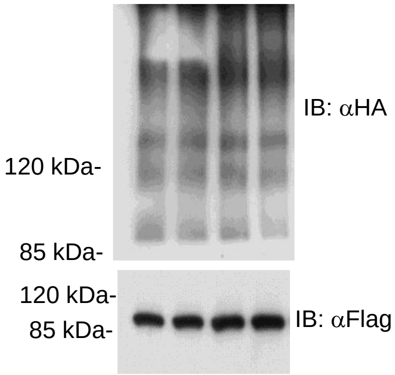

Figure 8e

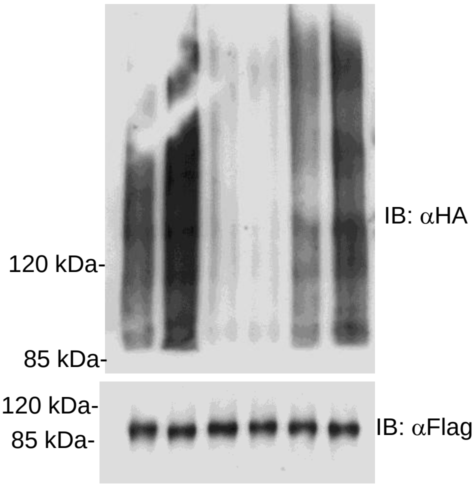

Supplementary Figure 12. Full size immunoblots of cropped blots in the main manuscript figures. Full size immunoblots of cropped blots for Fig. 8c-e.

Supplementary Fig. 1e

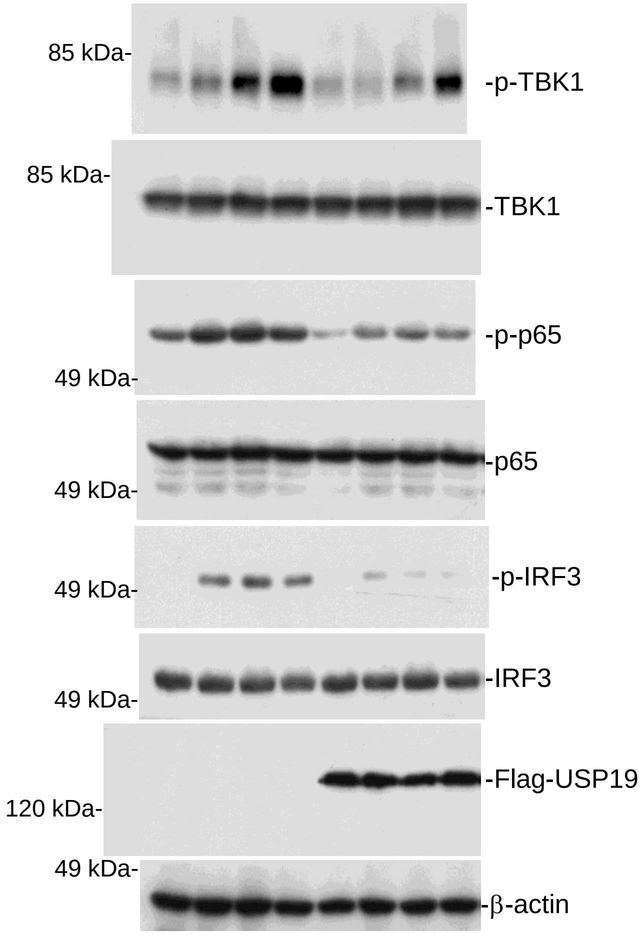

Supplementary Fig. 3c

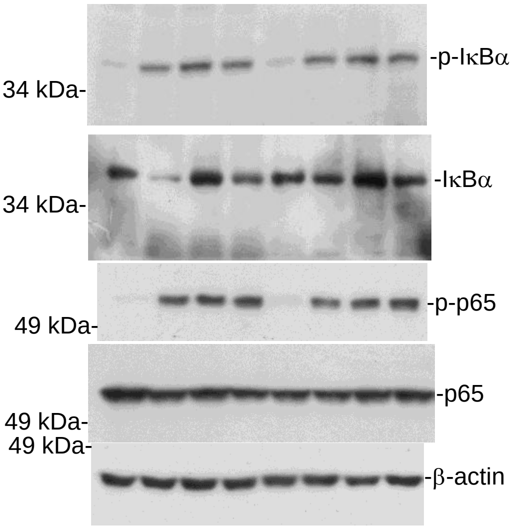

Supplementary Fig 5e

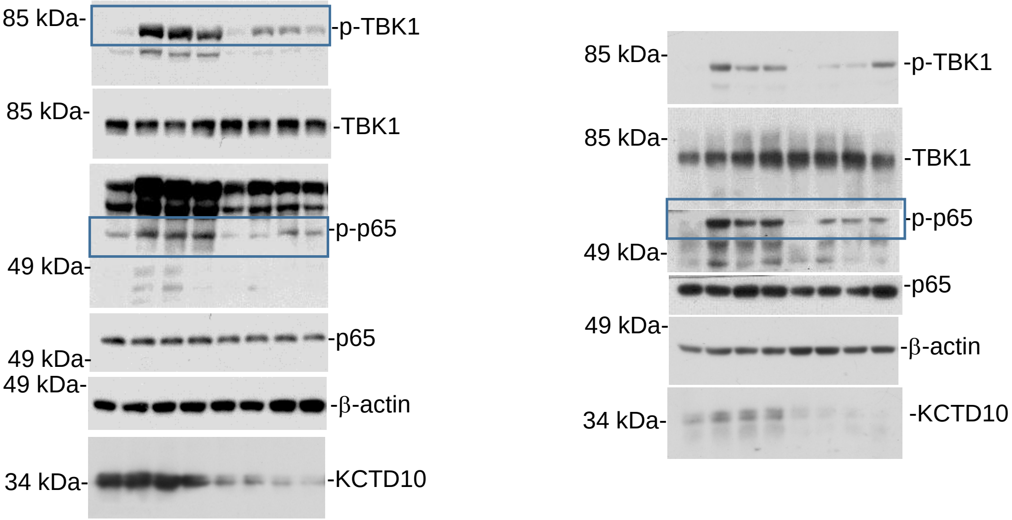

Supplementary Figure 13. Full size immunoblots of cropped blots in the main manuscript figures.
Full size immunoblots of cropped blots for supplementary Fig. 1e, 3c and 5e.

Supplementary Table 1. qPCR primer sequences

Gene	Forward	Reverse
GAPDH	GACAAGCTTCCCGTTCTCAG	GAGTCAACGGATTTGGTCGT
IFNB1	TTGTTGAGAACCTCCTGGCT	TGACTATGGTCCAGGCACA
CXCL10	GGTGAGAAGAGATGTCTGA	GTCCATCCTTGGAAGCACT
ISG56	TCATCAGGTCAAGGATAGTC	CCACACTGTATTTGGTGTCT
TNF	GCCGCATCGCCGTCTCCTAC	CCTCAGCCCCCTCTGGGGTC
USP19	CTGGTGAGTGTCAGCAAGGA	GAGGGCAAGAAAACACGGTG
IRF1	GAGGAGGTGAAAGACCAGA	TAGCATCTCGGCTGGACTTCG
TRIF	ACCTTCTGCGAGGATTTCCA	CGACAGTCGAAGTTGGAGGT
Gapdh	ACGGCCGCATCTTCTTGTGCA	ACGGCCAAATCCGTTCACACC
Ifnb1	TCCTGCTGTGCTTCTCCACCA	AAGTCCGCCCTGTAGGTGAGG
Cxcl10	ATCATCCCTGCGAGCCTATCCT	GACCTTTTTTGGCTAAACGCTT
Isg56	ACAGCAACCATGGGAGAGAAT	ACGTAGGCCAGGAGGTTGTGC
Il6	TCTGCAAGAGACTTCCATCCA	AGCCTCCGACTTGTGAAGTGG
Tnf	GGTGATCGGTCCCCAAAGGGATGA	TGGTTTGCTACGACGTGGGCT
